# COVID-19 Vaccination Attitudes With Neuromyelitis Optica Spectrum Disorders: Vaccine Hesitancy and Coping Style

**DOI:** 10.3389/fneur.2021.717111

**Published:** 2021-08-06

**Authors:** Yafang Xu, Yanpei Cao, Yue Ma, Yan Zhao, Hong Jiang, Jiahong Lu, Chongbo Zhao, Chao Quan

**Affiliations:** ^1^Department of Nursing, Huashan Hospital, Fudan University, Shanghai, China; ^2^Department of Neurology, Huashan Hospital, Fudan University, Shanghai, China

**Keywords:** COVID-19 vaccination attitude, vaccine hesitancy, coping style, neuromyelitis optica spectrum disease, COVID-19 vaccination

## Abstract

**Background:** Vaccination is an important method by which to stop the spread of coronavirus disease 2019 (COVID-19) in a population. Patients with neuromyelitis optica spectrum disorders (NMOSD) have unstable immune function and receive immunosuppressive therapy frequently, so they are hardly to make a decision to receive vaccination. Our study investigated the vaccine hesitancy and coping styles in patients with NMOSD to analyze the relationship between vaccine hesitancy and coping styles, and elucidate the factors influencing vaccine hesitancy.

**Methods:** A convenient sampling method was used to recruit participants. The Adult Vaccine Hesitancy Scale and Medical Coping Modes Questionnaire were used to measure the vaccine hesitancy and coping style of the participants. Pearson correlation, multiple stepwise, linear regression, and one-way analysis of variance were used to analyze the data.

**Results:** A total of 262 NMOSD patients were investigated. The score of vaccine hesitancy in NMOSD patients is lower (21.13 ± 4.355) than 25 points which indicated the patient is not considered to have vaccine hesitancy. The score for vaccine hesitancy was negatively correlated with the confrontation and avoidance coping styles (*r* = −0.481 and *r* = 0.423). That adoption of the coping styles of confrontation and avoidance as well as the residence of the patient were predictors of vaccine hesitation in NMOSD patients (*R*^2^(adj) = 0.29, *P* < 0.001). In addition, the vaccine hesitancy scores of NMOSD patients residing in rural areas were significantly higher than those of patients living in urban areas (*P* < 0.01). Comparing with each level of education, the scores were not statistically significant in vaccine hesitancy and coping styles (*P* > 0.05).

**Conclusions:** This study reveals that the NMOSD patients is not considered to have vaccine hesitancy, Patients who tend to adopt confrontation and avoidance coping styles have less vaccine hesitancy. Health authorities and medical specialist teams should strengthen effective vaccination information for patients with NMOSD, such as expert consensus or guidelines through various media to help them with decision-making. The significance of vaccination, the safety and side effects of COVID-19 vaccination and predicting of epidemiological trends of COVID-19 should be emphasized. More attention should be paid to NMOSD patients who living in rural areas.

## Introduction

The coronavirus 2019 disease (COVID-19) is an infection disease caused by the severe acute respiratory syndrome coronavirus 2 (SARS-CoV-2). COVID-19 pandemic is become the most serious global public health and has received widespread attention. Vaccination has been proven to be an important method to stop the spread of SARS-CoV-2 (1, 2). The World Health Organization stressed that COVID-19 vaccination could provide benefits to the community in the acute period of the pandemic (3–5). The effects of different types of vaccine to against SARS-CoV-2 were reported (6), the patients using immunosuppressive therapies may be at greater risk for COVID-19 due to their abnormal immune status (7, 8).

Neuromyelitis optica spectrum disorder (NMOSD) is a chronic inflammatory demyelinating disease. Because of their abnormal immune function, patients with these conditions often are at a high risk of COVID-19 infection (9, 10). The capability of the immune system to resist viral infection is based on the humoral response mediated by B-cells. Some studies have shown B-cell inhibitors such as rituximab and azathioprine are often used as a long-term treatment method for the patients with NMOSD (11–14), which could reduce concentrations of immunoglobulins G and M and increase the risk of COVID-19 infection (9, 15). Therefore, patients who developed iatrogenic lymphopenia should be advised to maintain social distancing even in areas where lockdown has been removed or ameliorated (16).

Vaccine hesitancy is one of the most significant health threats worldwide (17). It is defined as the delay in or refusal of vaccination when vaccination services are available. Although vaccination can effectively reduce the risk of contracting COVID-19, vaccine hesitancy persists among people due to worries about the safety of the vaccines. Patients with NMOSD may hesitate to receive COVID-19 vaccines due to their abnormal immune status and level of infection risk, which will adversely affect the vaccination rate in this population.

Coping styles are defined as a group of individual behaviors and physiological characteristics (18). Good coping styles result in healthy decision-making, better mental health, and improved quality of life (19); it is helpful to establish health protection behaviors and positive emotions and coping strategies to help alter the outcomes of disease (20, 21). Differences in COVID-19 vaccination attitudes among patients may lead to variable coping strategies and attitudes toward COVID-19.

This study aimed to investigate the vaccine hesitancy and coping styles in patients with NMOSD and to analyze the relationship between vaccine hesitancy and coping styles, seeking to elucidate the factors influencing vaccine hesitancy.

## Materials and Methods

### Participants

A total of 262 patients with NMOSD were recruited from the “NMO-MS Shanghai family” group (www.nmofamily.cn) affiliated with the Shanghai Rare Disease Support Foundation from February to April 2021. All participants were from 22 provinces geographically dispersed across China. The medical history and diagnosis of each participant were reviewed by neurologists at Huashan Hospital. The inclusion criteria of this study were as follows: (1) the diagnosis matched the NMOSD diagnosis criteria established by an international panel; (2) the prospective participant had normal cognitive function; and (3) the participant had the capacity to read, listen to, understand, and complete the questionnaire. Study exclusion criteria were as follows: (1) current immunosuppressant therapy for the treatment of another disease and (2) the presence of another chronic disease, such as cancer or diabetes. 230 patients completed the online questionnaire and 32 patients were investigated at the demyelinating disease outpatient clinic at Huashan Hospital. We used an online questionnaire to investigate the level of vaccine hesitancy and coping styles of each participant.

### Ethical Considerations

This study was reviewed by the ethics review committee of Huashan Hospital, affiliated with Fudan University, and met the ethical standards of the Declaration of Helsinki. Each patient signed a written informed consent form before their inclusion.

### Questionnaire Instruments

#### Adult Vaccine Hesitancy Scale

The adult vaccine hesitancy scale is usually used to measure vaccine hesitancy in an adult population (22) and was developed from vaccine hesitancy scale by Peretti-wattle et al. in 2015 (23). It has demonstrated good reliability and validity in one study assessing the hesitation and attitudes toward COVID-19 vaccination among American adults, with a Cronbach's α coefficient of 0.893. Lu et al. (22) confirmed the reliability and validity of the Chinese mainland version of the questionnaire, with a Cronbach's α coefficient of 0.729, and then used it to investigate the attitudes of the Chinese mainland population toward COVID-19 vaccination. The adult vaccine hesitancy scale has two dimensions and 10 items: the lack of confidence dimension contains even items (items 1, 2, 3, 4, 6, 7, and 8) and the risk dimension contains three items (items 5, 9, and 10). Possible answers range from least hesitant (one point) to most hesitant (five points). If the score is higher than 25 points, the respondent is considered to have vaccine hesitancy (22). Peretti-Wattel et al. defined the lack of self-confidence and risk dimensions of the vaccine hesitancy scale as “the level of confidence in health authorities and mainstream medicine” and “healthism/risk culture” (23). To date, the adult vaccine hesitancy scale has been used to investigate adult attitudes toward COVID-19 vaccination in the United States, China, Malaysia, New Zealand, and other countries (22).

#### Medical Coping Modes Questionnaire

The MCMQ was developed by Feifel et al. in 1991 (24) and is used to assess the styles of patients' coping with diseases; in particular, it is considered especially suitable for the assessment of coping styles of patients with chronic diseases. Shen and Jiang (25) translated into Chinese and revised the scale in 2000; this revised medical coping questionnaire has 20 items and three dimensions, where each dimension represents one coping style that patients may use to face diseases. The confrontation coping style dimension consists of items 1, 2, 5, 10, 12, 15, 16, and 19; the avoidance coping style dimension consists of items 3, 7, 8, 9, 11, 14, and 17; and the Acceptance-Resignation coping style dimension consists of items 4, 6, 13, 18, and 20. The Cronbach's α coefficients of each of these three dimensions are 0.69, 0.60, and 0.76. Confrontation coping styles can help patients to actively cope with their disease and seek treatment, while avoidance coping styles can help patients to divert attention from their disease so as to reduce their physical and mental symptoms and relieve psychological pressure. Finally, patients who adopt a resigned coping style are prone to experiencing pessimistic and negative emotions and often lose confidence in disease treatment (26). The MCMQ has been widely used in the psychosomatic research of patients with cancer, surgery, chronic hepatitis, and neurological diseases.

## Statistical Methods

Study data were analyzed using the Statistical Package for the Social Sciences version 24.0 software program (IBM Corporation, Armonk, NY, USA). Those data that were normally distributed are described as mean ± standard deviation values, while those that were not normally distributed relied on median (interquartile range) values for description, and enumeration data are described as percentages. We used partial correlation analysis to perform pairwise correlation analysis for each variable; a value of |r| from 0.00 to 0.19 suggests a very low correlation, that from 0.20 to 0.39 suggests a low correlation, that from 0.40 to 0.60 suggests a moderate correlation, that from 0.70 to 0.89 suggests a high correlation, and that from 0.90 to 1.00 suggests an extreme correlation. The factors influencing vaccine hesitation in NMOSD patients were analyzed by multiple stepwise linear regression equation and were compared by one-way analysis of variance.

## Results

### Characteristics of Patients With NMOSD

In this study, a total of 262 NMOSD patients were investigated. A total of 262 questionnaires were sent out and all of them were valid. Among 262 patients, 42 (16%) were male and 220 (84%) were female. Table 1 shows the characteristics of participants with NMOSD. The median age of the patients with NMOSD was 36 years, and the median duration of the disease was 4 years. Mycophenolate mofetil and oral steroids were most used as immunosuppressive therapies.

**Table 1:** Characteristics of study participants with NMOSD.

**No. of patients**		**262**	**Monthly household income**	**0-1999 RMB % (*N*)**	**14.5% (38)**
Age median (IQR)		36 (29, 46)		2000-3999 RMB % (N)	24.4% (64)
Gender	Male % (*N*)	16% (42)		4000-5999 RMB % (*N*)	25.2% (66)
	Female % (*N*)	84% (220)		6000-7999 RMB % (*N*)	14.1% (37)
Antibody	AQP4-IgG seropositivity % (*N*)	59.2% (155)		8000-9999 RMB % (*N*)	6.1% (16)
	MOG seropositivity % (*N*)	17.9% (47)		≥10000 RMB % (*N*)	15.6% (41)
	Both negative % (*N*)	22.9% (60)	Treatment	Mycophenolate mofetil % (*N*)	37.8% (90)
Course of disease	Median (IQR)	4 (2,5)		Rituximab % (*N*)	14.9% (39)
No. of relapse	Median (IQR)	2 (1,4)		Azathioprine % (*N*)	14.3% (35)
Location	Urban % (*N*)	71.8% (188)		Oral steroids	45% (118)
	Rural % (*N*)	28.2% (74)		Other immunosuppressants	24.4% (64)
Education	Primary school % (*N*)	46.6% (24)		No immunosuppressor	9.1% (24)
	High school % (*N*)	37.4% (98)	aVHS total score (M±SD)		21.13 ± 4.355
	University % (*N*)	47.3% (124)		Lack of confidence (M ± SD)	14.47 ± 3.595
	Post graduated % (*N*)	6.1% (16)		risk (M ± SD)	6.66 ± 1.396
Occupation	Student % (*N*)	9.2% (24)	MCMQ	confrontation (M ± SD)	19.10 ± 2.579
	worker % (*N*)	33.6% (88)		avoidance(M ± SD)	15.71 ± 2.125
	Retire % (*N*)	14.1% (37)		Acceptance-Resignation(M ± SD)	12.77 ± 1.197
	Unemployed % (*N*)	41.2% (108)			
Medical insurance	Medical insurance payment % (*N*)	24% (63)			
	Part of medical insurance payment % (*N*)	45.4% (119)			
	Self-paid % (*N*)	30.5% (80)			

*aVHS, adult vaccine hesitancy scale*.

*MCMQ, medical coping modes questionnaire*.

The results also shows that the total score of the aVHS in NMOSD patients was 21.13 ± 4.355 points, Following the interpretation of the aVHS score, if the score is lower than 25 points, the respondent is not considered to have vaccine hesitancy (22).

### Factors Influencing Vaccine Hesitancy Among NMOSD Patients

The Pearson correlation analysis was performed to analyze the correlation between vaccine hesitancy and coping style. The results showed that the total score of vaccine hesitancy and two dimensions were negatively correlated with the confrontation coping style to a moderate degree, and negatively correlated with the avoidance and acceptance-resignation coping style to a lower degree (Table 2).

**Table 2:** Pearon correlation analysis of vaccine hesitancy and coping style.

	**1. aVHS score**	**2. aVHS-confidence**	**3. aVHS-risk**	**4. MCMQ-confrontive**	**5.MCMQ-avoidance**	**6.MCMQ-Acceptance-resignation**
1	1					
2	0.957^**^	1				
3	0.652^**^	0.403^**^	1			
4	−0.481^**^	−0.423^**^	−0.412^**^	1		
5	−0.325^**^	−0.308^**^	−0.223^**^	0.204	1	
6	−0.110^**^	−0.122^**^	−0.131^**^	0.043	0.035	1

** *P < 0.01*.

*aVHS, adult vaccine hesitancy scale*.

*MCMQ, medical coping modes questionnaire*.

Multiple linear regression analysis was used to investigate the factors influencing vaccine hesitancy in NMOSD patients. The duration of disease, number of cases of disease relapse, urban/rural, education level, occupation, and scores of the three coping styles were used as independent variables, and the total score of vaccine hesitation was set as the dependent variable. A stepwise regression method was used, and *P* < 0.05 was used as the standard for screening variables. The result showed that the two of three total coping styles and urban/rural were the predictors of vaccine hesitation in NMOSD patients (Table 3).

**Table 3:** Stepwise regression analysis of factors influencing vaccine hesitancy in patients with NMOSD.

	**Standardized coefficient (β)**	***t***	**Adjusted *R*^**2**^**	***F***
MCMQ-confrontation	−0.404	−7.421	0.29	36.428^***^
MCMQ-avoidance	−0.239	−4.445		
Residence	0.131	2.469		

*** *P < 0.001*.

*MCMQ, medical coping modes questionnaire*.

### Influence of Urban/Rural on Vaccine Hesitancy and Coping Styles of NMOSD Patients

**Figure 1:**
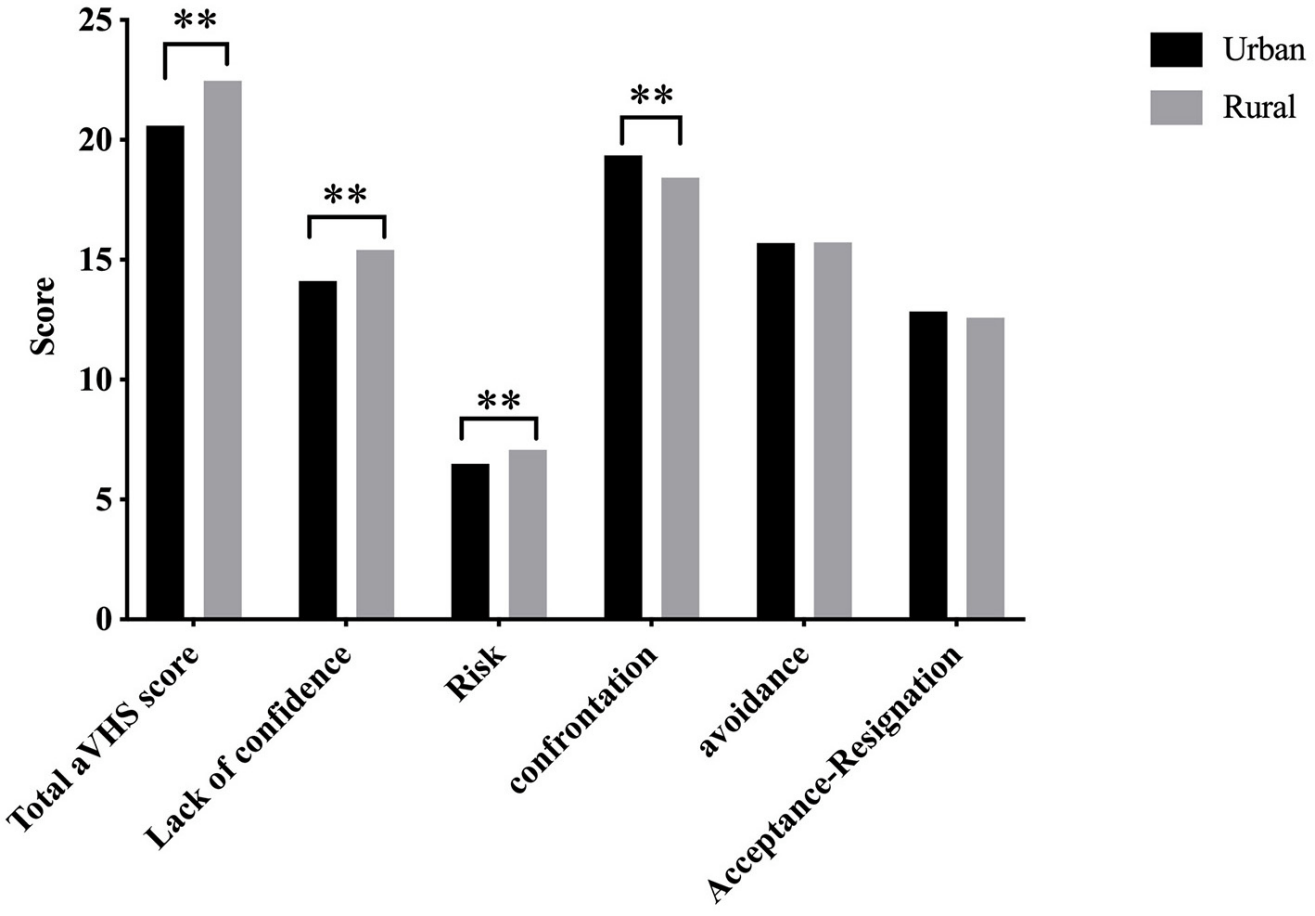
Comparison of vaccine hesitancy and coping styles of NMOSD patients according to urban/rural. **P < 0.01.

Base on the result of multiple linear regression analysis, One-way analysis of variance was used to further analyze the differences in vaccine hesitancy and coping styles of NMOSD patients in urban/rural. Compared with those in urban areas, patients living in rural areas had significantly higher total vaccine hesitation scores and scores of the three coping styles than those in urban areas, and the difference was statistically significant (*P* < 0.01). Regarding the confrontation coping style, the scores of patients in urban areas were significantly higher than those in rural areas, and the difference was statistically significant (*P* = 0.007) (Figure 1).

### Influence of Education on Vaccine Hesitancy and Coping Styles of NMOSD Patients

**Figure 2:**
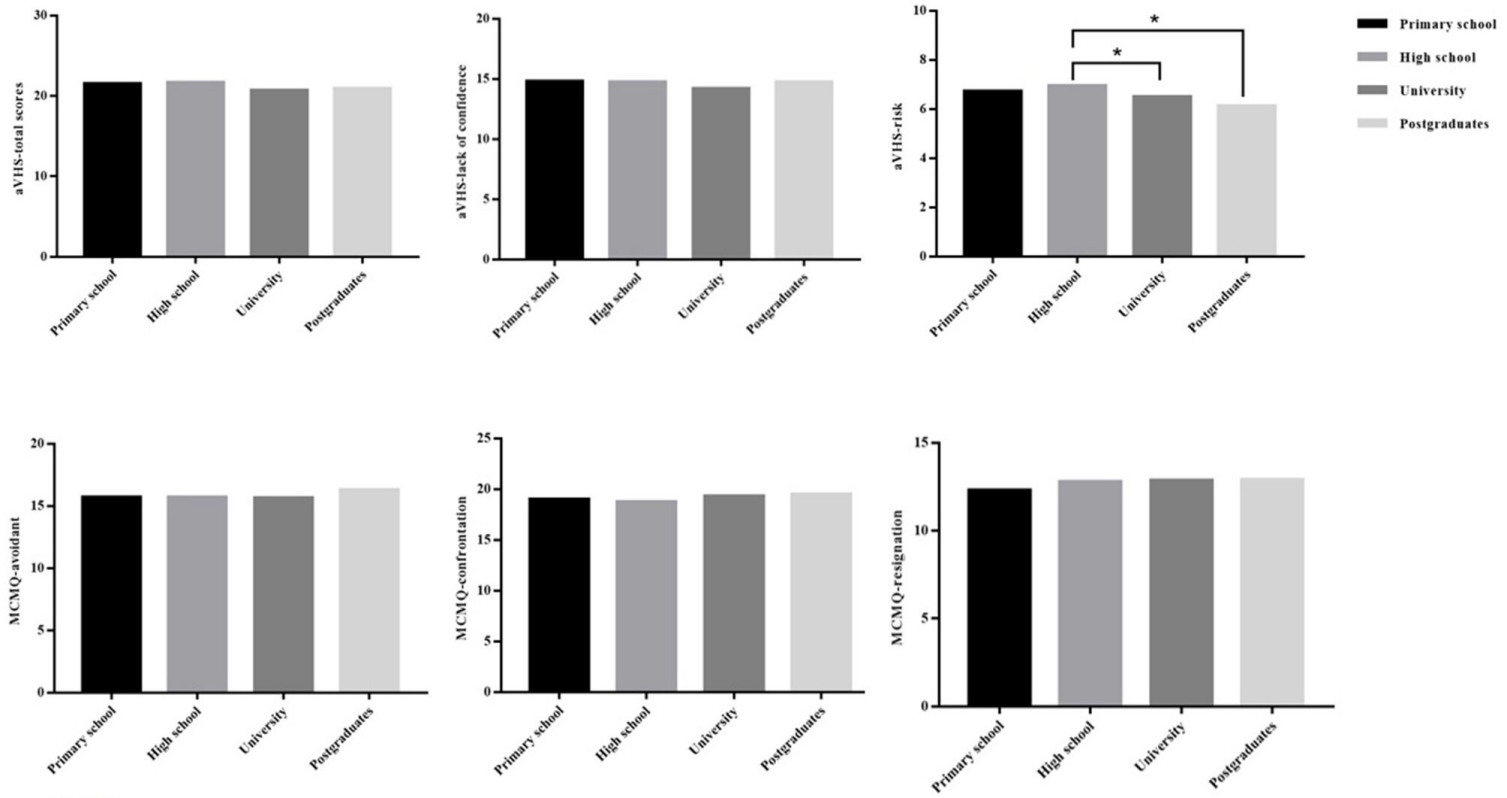
Comparison of vaccine hesitancy and coping styles of NMOSD patients according to level of education. *P < 0.05.

Although the results showed that education was not a predictor of vaccine hesitation in NMOSD patients, there are many literatures reported that education level could influence on vaccine hesitancy of individuals (27–31). One-way analysis of variance was used to analyze the differences in vaccine hesitancy and coping styles of NMOSD patients in different level of education. Compare with each level of education, the scores were not statistically significant in vaccine hesitancy and coping styles. However, the patients received high school education had a higher score compared to the patients received university or postgraduate education in risk dimension of aVHS, the difference was statistically significant (*P* = 0.043) (Figure 2).

### Characteristics of Patients With NMOSD Who Received COVID-19 Vaccination

Among the 262 patients with NMOSD, seven patients have received COVID-19 vaccination, which included six patients on immunosuppressant therapy and one patients not on medication (Table 4).

**Table 4:** Characteristics of NMOSD patients who received COVID-19 vaccines.

**No. of patients**		**7**	**Residence**	**Urban % (*N*)**	**57.1% (4)**
Age Median (IQR)		39(39, 57)		Rural % (*N*)	42.9 % (3)
Gender	Male % (*N*)	14.2% (1)	Treatment	Mycophenolate mofetil % (N)	28.5 % (2)
	Female % (*N*)	85.8 % (6)		Azathioprine % (*N*)	14.3 % (1)
Education	Primary school % (*N*)	28.6 % (2)		Oral steroids	42.9 % (3)
	High school % (*N*)	57.1% (4)		No immunosuppressor	14.3 % (1)
	University % (*N*)	14.3 % (2)			
	Postgraduates % (*N*)	0	aVHS score (M ± SD)		18.71 ± 2.812
Course of disease	Median (IQR)	5 (4, 5)		aVHS-confidence	12.43 ± 2.370
No. of relapse	Median (IQR)	2 (2,4)		aVHS-risk	6.29 ± 1.380
Occupation	Student % (*N*)	0	MCMQ (M ± SD)	MCMQ-confrontive	21.14 ± 3.716
	worker % (*N*)	57.1% (4)		MCMQ-avoidant	16.57 ± 2.225
	Retire % (*N*)	42.9 % (3)		MCMQ-resignation	12.14± 1.574
	Unemployed % (*N*)	0			

*aVHS, Adult vaccine hesitancy scale*.

*MCMQ, medical coping modes questionnaire*.

## Discussion

Health workers should understand that addressing vaccine hesitancy has become a priority of the global vaccine action plan. The World Health Organization Strategic Advisory Group of Experts (SAGE) on Immunization also set up a special workgroup to solve the problem of vaccine hesitancy (32). The SAGE vaccine hesitancy workgroup proposed that it is necessary to investigate the vaccine hesitancy of a population both before and during vaccination (23). Due to the lack of support from large-scale clinical trial results, the safety of vaccination for patients with demyelinating diseases is still controversial, so the attitude of patients toward vaccination is very important. Based on the investigation of vaccine hesitation in this study, it was found that NMOSD patients intend to acceptance of COVID-19 vaccines. The dimensions of the lack of self-confidence and risk reflect the level of confidence in health authorities and mainstream medicine and the health/risk culture of the person. Thus, we considered that NMOSD patients put their trust in mainstream medicine and health authorities.

Receiving vaccination is a self-protection measure and a decision made by an individual to protect them from COVID-19. People who have vaccine hesitancy often have no clear understanding of vaccination issues; they may lack knowledge but are very interested in vaccination and are determined to seek information and conduct long-term balanced decision-making (23). According to the theory of protective decision-making, before making protective behavior decisions, individual cognition will involve a series of decisions as follows: (1) risks are identified and assessed, that is, “do I need to take protective measures?”; (2) protective measures are searched for, that is, “what can one do to protect themselves?”; (3) protective measures are implemented, that is, “do protective measures need to be taken now?”; and (4) communication actions are assessed, that is, “where and how do I get this information?” The response to an individual's behavior, such as using various coping styles to solve problems, is one of the primary methods for individuals to make decisions (33).

The results showed a correlation between vaccine hesitancy and coping style. Patients who use confrontation, avoidance, and Acceptance-Resignation coping styles are more likely to accept COVID-19 vaccination and, among the three coping styles, the coping style of confrontation is the most relevant one. This study also investigated factors influencing vaccine hesitancy in patients with NMOSD. The results showed that adoption of the coping styles of confrontation and avoidance as well as the residence of the patient's residence were predictors of vaccine hesitation in NMOSD patients, that patients living in rural areas had more vaccine hesitancy than those living in urban areas. When comparing the total scores of vaccine hesitancy and the dimensions of the lack of self-confidence and risk, patients living in rural areas had significantly higher scores than those in urban areas. Our results revealed Patients with NMOSD who adopted a confrontation and avoidance coping style were more likely to accept COVID-19 vaccination.

Education was considered as a factor to influence the vaccine hesitancy in previous study. Some studies reported that lower level of education was significantly associated with against vaccination (27–29). But the finding of other studies reported the opposite opinion, the higher level of education was related to vaccine hesitancy (30, 31). In this study, the results indicated that the education did not significantly associated with vaccine hesitancy and coping styles in patients with NMOSD. However, we also found that the patients who received university or above education reported lower risk perception of vaccine hesitancy than those received high school education. The dimension of aVHS (risk) investigated the concern about safety and side effects of vaccine, the viewpoint about some vaccines no longer needed (the disease which need vaccination to prevent no longer common) (34), thus, we should put more attention on these points.

Among the seven patients who received vaccines, six patients were on immunosuppressants before and during vaccination. According to data from the United States Centers for Disease Control and Prevention, side effects such as injection site pain, fatigue, headache, muscle pain, fever, joint pain, chills, nausea, and swelling were reported by recipients of the Pfizer–BinNTech vaccine (32). In this study, all seven patients received a Chinese-made COVID-19 vaccine, they all reported similar side effects to those reported in the United States.

### Clinical Implications

As a positive factor, the confrontation coping style has an active impact on the patient's disease treatment and rehabilitation (35). Patients who use positive coping styles may seek various methods of disease treatment actively and are quick to accept doctors' recommendations for disease treatment, always showing good compliance with treatment (35, 36). Most of the confrontation coping style items were associated with the patient's request for disease-related knowledge from public media and medical staff (24) (Item 5. “In the past few months, how much knowledge about the disease have you obtained from doctors, nurses, and other health workers?” Item 10. “Do you often ask the doctor what you should do about your disease?” Item 12. “How much information have you learned about your disease from books, magazines, and newspapers in recent months?” Item 15. “How many questions did you ask the doctor about the disease?”) and are based on the theoretical model of protection decision-making, we suggest that an expert consensus or guidelines from medical consultants about COVID-19 vaccination for NMOSD patients should be developed and published by official media.

The patients who used avoidance coping style was reported that they tend to deny the risk of disease (36), vaccination might be considered as a prevention measure to avoid suffering from COVID-19 for the patients adopted avoidance coping style. Therefore, the doctors give the patients information actively about the significance of vaccination could help patients reduce the vaccine hesitancy.

We suggest that the medical consultant should describe the details clearly in guidelines and expert consensus about the significance of vaccination, safety, and the side effects of COVID−19 vaccine, predicting the epidemiological trends of COVID-19 could explain the necessity of vaccination to the NMOSD patients. These ways would be helpful for the patients to establish correct attitudes and beliefs about vaccination and to make correct decisions. Health authorities and medical and nurse specialists should pay more attention to NMOSD patients living in rural areas, as effective popular information dissemination of vaccines for patients with NMOSD could enhance their knowledge of diseases and vaccination.

### Limitations

Although the result was shown that NMOSD patients didn't have vaccine hesitancy, there was a limited number of patients who received COVID-19 vaccines in this study. This condition might be associated with the proceeding of the COVID-19 vaccination in China. The survey of this study began from February to April in 2021, the government was gradually expanding the population to vaccinate at that time, most of the patients in the study were waiting for the notification of COVID-19 vaccination by medical facility. In the future, we will follow up these patients to observe the number of vaccinated and verify the validation of our findings.

## Conclusion

This study reveals that the NMOSD patients is not considered to have vaccine hesitancy, they put their trust in mainstream medicine and health authorities. Patients with NMOSD who tend to adopt confrontation and avoidance coping styles toward vaccination. Health authorities and medical specialist teams should strengthen the dissemination of vaccination-related knowledge for patients such as an expert consensus or guidelines through various media. Some key points should be emphasized in the knowledge about vaccination, such as the significance of vaccination, the safety and side effects of COVID-19 vaccination and predicting of epidemiological trends of COVID-19. These points can help patients to make a decision and choose the right time for their vaccination. More attention should be given to the NMOSD patients living in rural areas to increase their knowledge of disease and vaccination.

## Data Availability Statement

The original contributions presented in the study are included in the article/supplementary material, further inquiries can be directed to the corresponding author/s.

## Author Contributions

YX and YC: writing. YM and YZ: data analysis. JL and HJ: reviewed and revised the manuscript. CZ and CQ: designed study and revised the manuscript. All authors contributed to the article and approved the submitted version.

## Conflict of Interest

The authors declare that the research was conducted in the absence of any commercial or financial relationships that could be construed as a potential conflict of interest.

## Publisher's Note

All claims expressed in this article are solely those of the authors and do not necessarily represent those of their affiliated organizations, or those of the publisher, the editors and the reviewers. Any product that may be evaluated in this article, or claim that may be made by its manufacturer, is not guaranteed or endorsed by the publisher.

## Abbreviations

COVID-19: Corona Virus Disease 2019
SARS-CoV-2: Severe Acute Respiratory Syndrome- Corona Virus-2
NMOSD: neuromyelitis optica spectrum disorder
aVHS: Adult Vaccine Hesitancy Scale
MCMQ: Medical Coping Modes Questionnaire.
